# Increased toxicity of a trinuclear Pt-compound in a human squamous carcinoma cell line by polyamine depletion

**DOI:** 10.1186/1475-2867-12-20

**Published:** 2012-05-28

**Authors:** Stina M Oredsson, Johan Wennerberg

**Affiliations:** 1 Department of Otorhinolaryngology/Head and Neck Surgery, University Hospital, S-221 85, Lund, Sweden; 2 Department of Oncology, University Hospital, S-221 85, Lund, Sweden; 3 Ferring Pharmaceuticals A/S, DK-2300, Copenhagen S, Denmark; 4 Department of Cell and Organism Biology, Lund University, S-223 62, Lund, Sweden

**Keywords:** Cisplatin, BBR3464, Head and neck carcinoma, α-difluoromethylornithine, *N*^1^,*N*^11^-diethylnorspermine, Isobologram, Polyamines

## Abstract

**Background:**

Mononuclear platinum anticancer agents hold a pivotal place in the treatment of many forms of cancers, however, there is a potential to improve response to evade resistance development and toxic side effects. BBR3464 is a promising trinuclear platinum anticancer agent, which is a polyamine mimic. The aim was to investigate the influence of polyamine pool reduction on the cytotoxic effects of the trinuclear platinum complex BBR3464 and cisplatin. Polyamine pool reduction was achieved by treating cells with either the polyamine biosynthesis inhibitor α-difluoromethylornithine (DFMO) or the polyamine analogue *N*^1^,*N*^11^-diethylnorspermine (DENSPM).

**Methods:**

A human squamous cell carcinoma cell line, LU-HNSCC-4, established from a primary head and neck tumour was used to evaluate cellular effects of each drug alone or combinations thereof. High-performance liquid-chromatography was used to quantify intracellular polyamine contents. Inductively coupled mass spectroscopy was used to quantify intracellular platinum uptake. Cells were exposed to DFMO or DENSPM during 48 h at concentrations ranging from 0 to 5 mM or 0 to 10 μM, respectively. Thereafter, non-treated and treated cells were exposed to cisplatin or BBR3464 during 1 h at concentrations ranging from 0 to 100 μM. A 96-well assay was used to determine cytotoxicity after five days after treatment.

**Results:**

The cytotoxic effect of BBR3464 on LU-HNSCC-4 cells was increased after cells were pre-treated with DENSPM or DFMO, and the interaction was found to be synergistic. In contrast, the interaction between cisplatin and DFMO or DENSPM was near-additive to antagonistic. The intracellular levels of the polyamines putrescine and spermidine were decreased after treatment with DFMO, and treatment with DENSPM resulted in an increase in putrescine level and concomitant decrease in spermidine and spermine levels. The uptake of BBR3464 was significantly increased after pre-treatment of the cells with DFMO, and varied dependent on the concentration of DENSPM. The uptake of cisplatin was unchanged.

**Conclusions:**

Taken together, these results demonstrate that combinations of polyamine synthesis inhibitors with BBR3464 appear to be a promising approach to enhance the anticancer activity against HSCC.

## Introduction

Although mononuclear platinum anticancer agents hold a pivotal place in the treatment of many forms of cancers, there is a potential to improve response and survival in patients. Development of resistance to therapy and toxic side effects are major problems, which have prompted research into new platinum drugs, displaying different mechanisms of action. One such compound is BBR3464, which is a promising trinuclear platinum anticancer agent. It is built up by three square-planar platinum units and it forms different types of adduct with DNA [1]. The structure of BBR3464 classifies it as a polyamine analogue. Based on the result from a screening panel with 60 cell lines, the mechanism of action of BBR3464 has been suggested to differ compared to cisplatin [2]. Furthermore, BBR3464 has been shown to be more potent than cisplatin [2,3] and to circumvent the resistance to cisplatin in a number of tumour cell lines and xenografts [4-7]. BBR3464 has also showed superior activity against p53-mutant tumours [8].

A fruitful way of increasing the response rate of tumours is to use complementary combinations of drugs, e.g. an antimetabolite and one or more anticancer agents. The platinum anticancer drugs cisplatin and oxaliplatin have been successfully combined with e.g. 5-fluorouracil (5-FU) in head and neck and colon cancer, respectively [9]. The combination of cisplatin or carboplatin with paclitaxel is the most common first-line treatment for patients with advanced ovarian cancer. For non-small-cell lung cancer combination chemotherapy involving platinum agents is considered to be important, however, limited by toxicity and resistance [10]. BBR3464 was successfully combined with 5-FU against a human gastric tumour model in preclinical *in vivo* studies. However, a phase I open-label dose-escalating study could not support administration of BBR3464 in combination with protracted venous infusion of 5-FU [11].

Naturally occurring polyamines have been recognized as important constituents for the proliferation of normal and malignant cells. Thus, the polyamine metabolic pathway has been an attractive target for the treatment of cancer. This has resulted in the development of a wide range of inhibitors of polyamine biosynthesis, the most thoroughly investigated being α-difluoromethylornithine (DFMO) [12]. Treating cells with DFMO, which irreversibly binds to the polyamine biosynthetic enzyme ornithine decarboxylase (ODC), reduces the polyamine levels resulting in inhibition of cell proliferation *in vitro*[12,13]. Another group of substances interfering with polyamine metabolism are polyamine analogues. *N*^1^*N*^11^-Diethylnorspermine (DENSPM) is a spermine analogue, which is taken up by the cellular polyamine transport system and thus is accumulated in the cell [14-16]. Intracellular DENSPM accumulation results in activation of the polyamine catabolic enzyme spermine/spermidine *N*^1^-acetyltransferase (SSAT) resulting in the catabolism of the natural polyamines putrescine, spermidine and spermine [14-16]. DENSPM can however not take over the function of the natural polyamines. In addition to stimulating catabolism, DENSPM also exerts a feedback inhibition of polyamine biosynthesis. DENSPM treatment as DFMO treatment results in inhibition of cell proliferation and has been demonstrated to induce apoptosis [17,18].

Cellular polyamine homeostasis is a very complex process involving biosynthesis, catabolism and transport through the cell membrane [19]. Thus, when cells are depleted of their natural polyamines, the polyamine transport system is up-regulated as a means for the cell to normalize its polyamine pools [19-21]. Since polyamines are continuously supplied from the gastrointestinal tract through food and bacteria, this is a major obstacle for effective inhibition of tumour-cell growth *in vivo* by use of biosynthesis inhibitors as single chemotherapeutic agents. However, an up-regulated polyamine transport system may be exploited in the search for new chemotherapeutic drugs. Cisplatin has been combined with inhibitors of the polyamine biosynthetic pathway with contradictory anti-tumour efficacies [22-28].

BBR3464 is a new polyamine cisplatin analogue [1]. Because of this, we hypothesise that BBR3464 is, at least to some extent, transported into the intracellular compartment by the same transport system as the naturally occurring polyamines. Based on that hypothesis, we investigated the antitumour effect of BBR3464 in combination with DFMO or DENSPM against a squamous carcinoma cell line of the head and neck (HNSCC), and compared that with cisplatin.

## Results

### Effect of DFMO and DENSPM on polyamine levels

**Figure 1:**
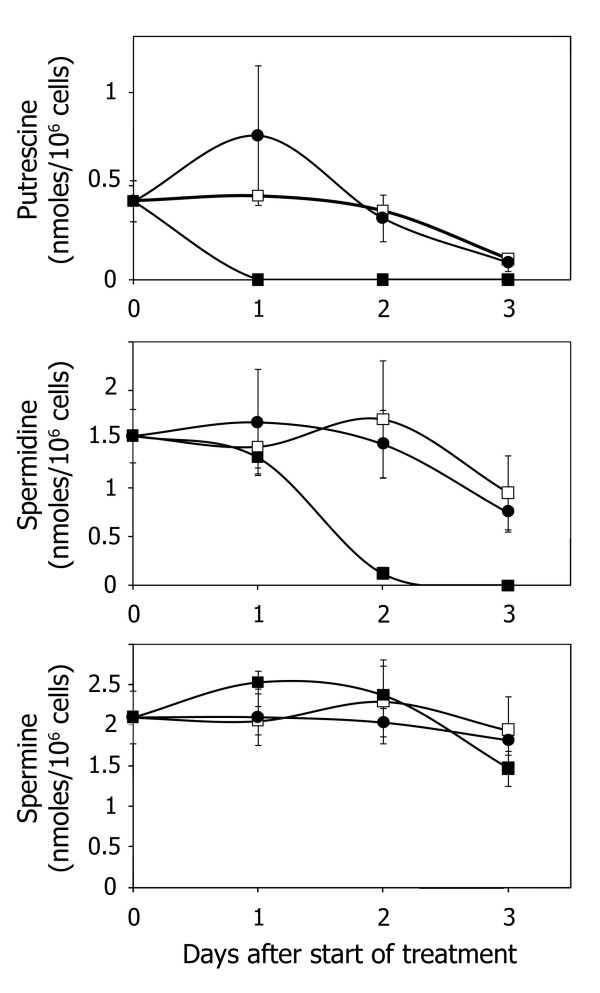
**The effect of 50 μM DFMO or 0.1 μM DENSPM treatment on the polyamine content of LU-HNSCC-4 cells.** The cells were seeded and incubated for 24 h before addition of DFMO or DENSPM at time 0 in the figure. The symbols are the means from one experiment with n = 3 and bars represent ± SD. Control cells (□); DFMO-treated cells (■); DENSPM-treated cells (●).

Treatment with 0.1 μM DENSPM or 50 μM DFMO affected the polyamine pools as have been previously observed [12]. DFMO treatment reduced the putrescine content to an undetectable level already after 24 h of treatment (Figure 1). The spermidine pool was almost depleted by DFMO treatment after 48 hours of treatment while the spermine pool was essentially unaffected. Treatment with DENSPM resulted in increased level of putrescine, and unchanged levels of spermidine and spermine (Figure 1). DFMO treatment reduced the total pool of polyamines. Based on these date we choose a 48 hours pre-treatment period with DENSPM or DFMO before treatment with cisplatin or BBR3464. 

### Effect of DFMO and DENSPM on cellular accumulation of cisplatin and BBR3464

The effect of DFMO or DENSPM treatment for 48 h on intracellular accumulation of cisplatin or BBR3464 after a 1 h exposure time was investigated. A significant increase in platinum content was found in cells that had been growing in the presence of 25 or 75 μM DFMO for 48 h before addition of BBR3464 (p < 0.002) as compared with cells grown in control medium (Table 1). The cellular level of BBR3464 was found to be slightly higher in cells that had been growing in the presence of 5 μM DENSPM for 48 h, while 10 μM DENSPM decreased the platinum accumulation as compared with control cells. When cisplatin accumulation was investigated, neither DFMO nor DENSPM were found to influence the amount of platinum in the cells (Table 1).

**Table 1:** **Effect of DFMO or DENSPM on intracellular accumulation of cisplatin and BBR 3464 in LU-HNSCC-4 cells.**^a^

Pre-treatment	Platinum accumulation (μg Pt/10^6^ cells)	
	Cisplatin	BBR 3464
Control medium	0.8 ± 0.1	1.0 ± 0.1
5 μM DENSPM	0.8 ± 0.1	1.2 ± 0.1
10 μM DENSPM	0.9 ± 0.1	0.7 ± 0.2
25 μM DFMO	0.9 ± 0.1	2.2 ± 0.3^*^
75 μM DFMO	0.8 ± 0.1	2.4 ± 0.4^*^

^a^Cells were grown in the absence of DENSPM or DFMO 48 h and then the medium was changed and fresh medium added before a 1 h exposure to cisplatin (7.5 μM) or BBR 3464 (0.5 μM). Values are mean from three independent experiments in each of which there were at least two independent samples  ±  SEM. ^*^ p   0.002.

### Cytotoxicity studies

**Figure 2:**
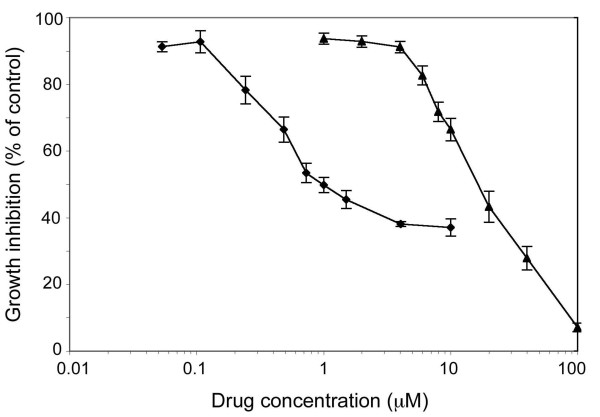
**Growth inhibition of LU-HNSCC-4 cells exposed to varying concentration of cisplatin (▲) or BBR3464 (♦).** Cells that had been grown in 96-well plates for 72 h were treated with the drugs at the indicated concentrations for 1 h. Thereafter the drug containing medium was removed and the cells incubated for 72 h before cell growth was evaluated with an MTT assay. Values represent averages of triplicate experiments, and error bars indicate SEM.

**Figure 3:**
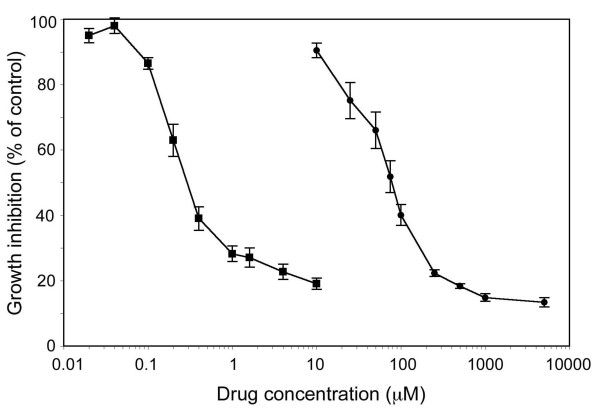
**Growth inhibition of LU-HNSCC-4 cells exposed to varying concentration of DFMO (**●**) or DENSPM (**■**).** Cells were seeded in 96-well plates and 24 hours later DFMO or DENSPM were added at the indicated concentrations. The cells were incubated with the drugs for 48 h. Thereafter the drug containing medium was removed and the cells incubated for 72 h before cell growth was evaluated with an MTT assay. Values represent averages of four or more experiments, and error bars indicate SEM.

The cytotoxic effect of a 1 h exposure to BBR3464 or cisplatin, or a 48 h exposure to DFMO or DENSPM, was investigated. BBR3464 was found to be one order of magnitude more cytotoxic than cisplatin (IC_50_: 1.2 *vs*. 17 μM, Figure 2). The dose-response curves for DFMO and DENSPM treatment alone for 48 h on the cell growth of LU-HNSCC-4 are shown in Figure 3. The IC_50_ concentrations of the two drugs were 80 μM DFMO and 0.32 μM DENSPM.

**Figure 4:**
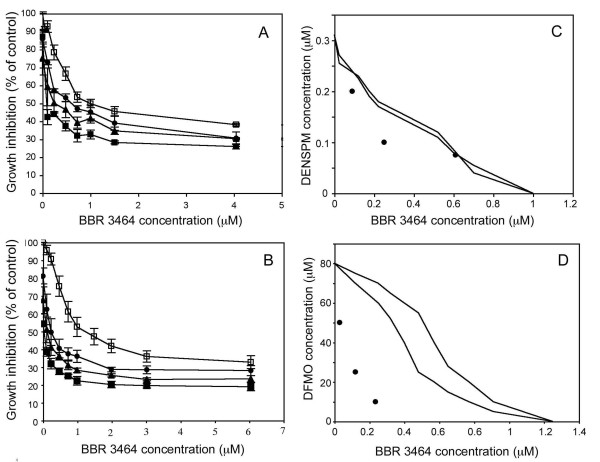
**Growth inhibition of LU-HNSCC-4 cells following treatment with BBR3464 alone or in combination with (A) DENSPM and (B) DFMO.** Cells were seeded in 96-well plates and 24 hours later DFMO or DENSPM were added at the indicated concentrations. The cells were incubated with the drugs for 48 h. Thereafter the cells were treated with BBR3464 at the indicated concentrations. After one 1 h of BBR3464 treatment, the drug containing medium was removed and the cells incubated for 72 h before cell growth was evaluated with an MTT assay. In the combination experiments cells were treated with different concentrations of DENSPM: 0 (□), 0.075 (●), 0.10 (♦), or 0.20 (■) μM, or DFMO: 0 (□), 10 (●), 25 (♦), or 50 (■) μM. Values represent average of triplicate experiments, and error bars indicate SEM. Isobolograms (**C**) and (**D**) for the combination of BBR3464 with DENSPM and DFMO, respectively.

In the combination experiments, we used concentrations of DFMO (10, 25, and 50 μM) or DENSPM (0.075, 0.10, and 0.20 μM) that had a   40% effect on cell growth on their own. When these concentrations of DFMO or DENSPM were combined with BBR3464, cell viability decreased compared with BBR3464 treatment alone, as illustrated in Figure 4. We found that the IC_50_ concentrations obtained from the drug combinations to be within or to the left of the envelope of additivity in the isobolograms, Figure 4, indicating additive to synergistic effects.

**Figure 5:**
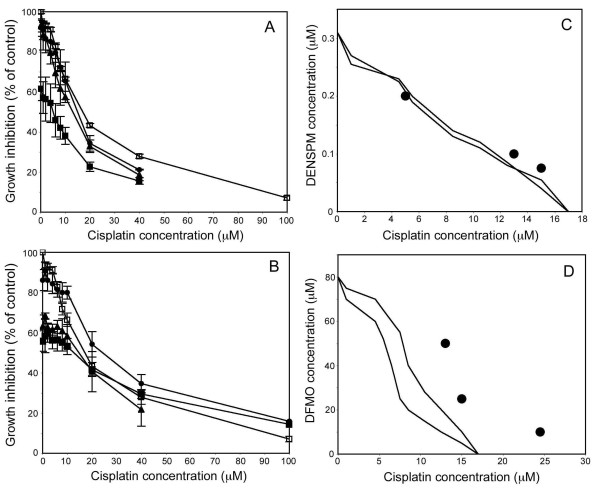
**Growth inhibition of LU-HNSCC-4 cells following treatment with cisplatin alone or in combination with DENSPM (A) and DFMO (B).** Cells were seeded in 96-well plates and 24 hours later DFMO or DENSPM were added at the indicated concentrations. The cells were incubated with the drugs for 48 h. Thereafter the cells were treated with cisplatin at the indicated concentrations. After one 1 h of BBR3464 treatment, the drug containing medium was removed and the cells incubated for 72 h before cell growth was evaluated with an MTT assay. In the combination experiments cells were treated with different concentrations of DENSPM: 0 (□), 0.075 (●), 0.10 (♦), or 0.20 (■) μM, or DFMO: 0 (□), 10 (●), 25 (♦), or 50 (■) μM. Values represent average of triplicate experiments, and error bars indicate SEM. Isobolograms (**C**) and (**D**) for the combination of cisplatin with DENSPM and DFMO, respectively.

When 0.075, 0.10, or 0.20 μM DENSPM was combined with cisplatin we found the cytostatic effect of cisplatin to increase, as illustrated in the dose-response curves in Figure 5. From these curves, the obtained IC_50_ concentrations were found to be near the envelope of additivity, indicating near-additive effects. When 10, 25, and 50 μM DFMO were combined with cisplatin, cell viability decreased for 10 and 25 μM DFMO and increased for 50 μM DFMO compared with cisplatin alone, as illustrated in the dose-response curves in Figure 5. The obtained IC_50_ values were found to be on the right of the envelope of additivity, indicating antagonistic to protective effects, Figure 5.

## Discussion

The results presented here indicate that combination of polyamine synthesis inhibitors with BBR3464, investigated in an *in vitro* HNSCC cell line established from a primary head and neck tumour, may be a potential regimen for head and neck cancer chemotherapy. A clear synergistic effect was observed when DFMO or DENSPM were combined with BBR3464. In contrast, for combinations with cisplatin the effect was only additive or antagonistic (protective in one case). Interestingly, since DENSPM and BBR3464 are polyamine analogues a competition in uptake and thus antagonistic effects could have been expected. However, it should be noted that the combination was sequential *i.e*. the cells were not treated simultaneously with the two compounds in any of the experiments. Thus, there should not be any competition in uptake between DENSPM and BBR3464.

Our study confirms previous results of higher antitumour effect of BBR3464 compared with cisplatin against *in vitro*-cultured tumour cell lines [2,4-8,29-31]. Several mechanisms for the higher antitumour effect of BBR3464 have been suggested, e.g. increased platinum binding to DNA, different types of DNA adducts, and that mechanisms involved in the repair of these adducts are different from those of cisplatin

Increased polyamine levels are associated with increased proliferation rate, decreased apoptosis and increased expression of genes affecting tumour invasion and metastasis [32]. Thus, the concept of treating cells with compounds that decrease the polyamine pools has been one approach to achieve inhibition of tumour growth. *In vitro*, the mechanism of action of DFMO, resulting in reduced cell proliferating, is mediated through inhibiting ODC, which results in depletion of putrescine and spermidine [12]. The decreased levels of putrescine and spermidine, and the reduced cell number observed here are in accordance with this mechanism. DENSPM, which is a spermine analogue, was developed to inhibit the activity of both ODC and *S*-adenosylmethionine decarboxylase. Furthermore, an unexpected effect of this analogue was the induction of the polyamine catabolic enzyme SSAT. The level of inhibition of cell proliferation by DENSPM is highly dependent on the level of induction of SSAT, which results in a massive depletion of the cellular polyamine pool [15]. Furthermore, it has been suggested that DENSPM replaces native polyamines at intracellular locations, without fulfilling their biological functions [33,34].

Here, we show that the intracellular levels of spermidine and spermine were reduced, while putrescine was increased compared to control after cells were exposed to DENSPM. These changes of the polyamine pools could be explained by the activated polyamine catabolism, resulting in decreased levels of spermidine and spermine coupled to increased levels of putrescine [35]. In our experimental setup, DENSPM was found to inhibit cell growth more compared with DFMO. This result is in accordance with earlier findings showing greater antiproliferative activity of DENSPM compared with DFMO [36]. DFMO treatment reduced the putrescine and spermidine pools while the spermine pool remained unchanged compared to control. In fact DFMO treatment slightly reduced the total polyamine pool while DENSPM treatment did not. Thus, it appears that the decreased spermine pool in DENSPM-treated cells was of importance for growth inhibition presumably together with the suggestion that DENSPM replaces native polyamine [33,34].

Although the growth of different tumour cells is suppressed by treatment with polyamine biosynthesis inhibitors and analogues, it has been recognized that tumours are able to regrow when administration of these compounds is interrupted [12]. Accordingly, polyamine biosynthesis inhibitors and analogues may be more useful as chemotherapeutic agents when combined with another drug. Indeed, sensitization of alkylating agents by DFMO and DENSPM, have been observed [22-24,37,38]. However, other studies have found only additive or antagonistic effects of combining cisplatin with polyamine synthesis inhibitors [28,37]. A possible explanation to the discrepancy between these observed results might be that the interaction of cisplatin and polyamine biosynthesis inhibitors seems to be schedule dependent [24]. However, it has also been proposed that DFMO-induced polyamine depletions alters the structure of DNA, which may impair the ability of cisplatinum to crosslink in the molecule [28].

In this study, we found a clear synergistic effect when DFMO or DENSPM were combined with BBR3464. In our experimental setup, cells were pre-treated with DFMO or DENSPM for 48 h prior to addition of the platinum complex. DFMO produced a larger synergistic effect compared with DENSPM, as the IC_50_ values are furthest to the left of the envelope of additivity in the isobolograms. A possible explanation for this may be related to the finding of a significantly higher platinum uptake in cells that were pre-treated with DFMO compared with DENSPM-treated and control cells. This change in cellular accumulation of BBR3464, as a result of DFMO or DENSPM treatment, suggests that the transport of BBR3464 in to the cell is mediated by the polyamine transport system. Interestingly, we found a decreased platinum uptake when cells were pre-treated with higher concentrations of DENSPM. This observation may reflect that DENSPM accumulates in the cell and that those native polyamines, associated with negatively charged molecules, e.g. DNA and RNA are replaced by the more inert DENSPM molecule. As a consequence, the ability of BBR3464 to enter into the cell passing through the polyamine transport system, and subsequently reach the intracellular targets may be reduced. Also, polyamine analogue treatment has been shown to induce antizyme, which would inhibit further uptake of polyamine like molecules since antizyme is an inhibitor of polyamine transport [12,39]. This may also contribute to the fact that DENSPM treatment at high concentrations reduces the uptake of BBR 3463. In contrast, DFMO does not substitute for the reduced levels of native polyamines. Instead, cells deprived of polyamines compensate by increasing the uptake of extracellular polyamines, thus facilitating accumulation of the cisplatinum polyamine analogue BBR3464 in the cellular compartment.

In contrast to BBR3464, cisplatin was not found to interact synergistically with DFMO or DENSPM. At best, near-additive effects were found for combinations with DENSPM, while antagonistic or protective effects were found for combinations with DFMO. Here, we found no difference in accumulation of cisplatin between cells pre-treated with DFMO or DENSPM and untreated cells. This finding is reasonable, since it has been demonstrated that tumour cell uptake of cisplatin is regulated through the major Cu influx transporter CTR1 [40], and thus, is not likely to be mediated by the polyamine transport system, as shown in a previous study [41].

Intriguing is the fact that very low concentrations of DFMO enhanced the uptake and toxicity of BBR3464. This is very promising from a cancer treatment point of view where a low dose of DFMO could increase the toxicity of BBR3464 substantially. DFMO has been used as a chemotherapeutic and chemopreventive agent in several studies with varying success [42,43]. Ongoing phase II and III studies show a favourable effect of combining DFMO and the non steroidal anti-inflammatory drug sulindac in the chemoprevention of colon cancer development in patients with colon adenomatous polyps [44].

## Conclusions

Our results argue that polyamine synthesis inhibitors and polyamine analogues may be useful in cancer chemotherapy in combination with BBR3464. Since there is large numbers of polyamine synthesis inhibitors available there is a rationale for continuous investigations along this avenue. Also, further experiments with BBR3464 in combination with polyamine synthesis inhibitors is needed to establish the effect on the growth of human tumours *in vivo*.

## Methods

### Drugs and chemicals

BBR3464 was generously provided by Dr Ernesto Menta from Novuspharma S.p.A. (Monza, Italy), now Cell Therapeutics Europe S.r.I. (Bresso, Italy), and was dissolved in saline immediately before use. Cisplatin was purchased from Pharmalink AB (Stockholm, Sweden). DENSPM was purchased from Tocris Cookson (Bristol, UK), and DFMO was purchased from Lonza Inc. (Allendale, NJ). DENSPM and DFMO were dissolved in phosphate-buffered saline (PBS, GIBCO, Paisly, Scotland) and the pH was adjusted to 7.4 with NaOH before use.

### Cell line and growth conditions

An established tumour line, LU-HNSCC-4, originating from HNSCC of the floor of the mouth was used in this study [45]. Cells were maintained in logarithmic growth as monolayer cultures in DMEM medium (GIBCO) supplemented with 10% heat-inactivated fetal calf serum and antibiotics (GIBCO) (100 units/ml penicillin and 100 units/ml streptomycin), in a humidified 5% CO_2_ atmosphere at 37°C.

### MTT assay

The MTT assay based on the tetrazolium dye was used to evaluate the effect of the drugs on cell growth [46]. Briefly, cells were harvested by trypsinization, electronically counted (NucleoCounter™, ChemoMetec A/S, Edison, NJ), seeded (1500 cells / well) in 96-well plates, and allowed to adhere overnight before addition of drug. In the case of treatment with DENSPM or DFMO alone, the drugs were added to medium and cells were incubated for 48 h at concentrations ranging from 0 to 10 μM and 0 to 5 mM, respectively. After removal of the medium containing DFMO or DENSPM, cells were allowed to regrow in drug-free medium for 72 h before the cell growth was determined using the MTT assay. In the case when cells were treated with cisplatin or BBR3464 alone, drugs were added in medium and cells were incubated for 1 h at concentrations ranging from 0 to 100 μM or 0 to 10 μM, respectively. Thereafter, the drug-containing medium was removed, and cells were allowed to regrow in drug-free medium for 72 h before cell growth was determined using the MTT assay.

The concentrations of DENSPM and DFMO chosen for the further studies described below were based on the results of the MTT assays described above.

In the combination experiments, cells were seeded in 96-well plates and allowed to adhere overnight. Then, cells were treated with DENSPM (0.075, 0.1, or 0.2 μM) or DFMO (10, 25, or 50 μM) during 48 h. Thereafter, the drug containing medium was removed, and cells were exposed for 1 h to cisplatin or BBR3464, at concentrations ranging from 0 to 100 μM or 0 to 10 μM, respectively. After drug removal, cells were allowed to regrow in drug-free medium for 72 h before the effect on cell growth was determined and the drug interactions were analysed by means of isobologram plotting, see below. The cytotoxicity experiments were performed in at least triplicate.

### Test for synergy

For each experiment, the IC_50_ values were determined as the concentration of drug causing a 50% growth inhibition compared to control. For each combination, the IC_50_ values were calculated, and the data from these experiments was analysed using an isobologram method [47]. In brief, the IC_50_-isobolgram shows the doses required to inhibit cell growth by 50% after exposure to cisplatin or BBR3464 alone, DENSPM or DFMO alone, or combinations thereof. In this analysis, it was taken into consideration that the cytostatic effect of each drug produced non-linear dose-response curves. Therefore, the area between the two lines in Figures 4 and 5 indicates the “envelope of additivity”. The results from each cell survival curve were merged and plotted. Dots located to the left, within, or to the right of the “envelope of additivity” indicate synergy, additivity and antagonism, respectively, between the two drugs.

### Intracellular accumulation

For the investigation of the intracellular accumulation of cisplatin and BBR3464, cells were seeded in tissue culture dishes (6 cm diameter) in triplicate and 24 hours later, they were exposed to DENSPM (5 or 10 μM) or DFMO (25 or 75 μM) or drug-free medium. Forty-eight hours after DENSPM or DFMO exposure, drug-containing medium was removed and cells were treated with 0.5 μM BBR3464 or 7.5 μM cisplatin for 1 h. The platinum accumulation was then analysed by inductively coupled plasma mass-spectrometry (Thermo X7, Thermo Elemental, Winsford, UK). All accumulation experiments were performed in at least duplicate and the experiment was repeated three times. Briefly, cells were washed three times with ice-cold PBS, harvested by trypsinization and counted and digested in 70% HNO_3_ for 2 h at 65°C. The samples were then diluted to a 2% acid solution before analysis. The samples were introduced in a segment-flow mode and analysed in peak-jumping mode, 100 sweeps and 1 point per peak, 30 ms dwell time for platinum (Pt^195^) and 10 ms dwell time for bismuth (Bi^209^) used as an internal standard. The detection limit calculated as three times the standard deviation (SD) of the blank was 0.02 μg/L. All samples were prepared in duplicate and the method imprecision, calculated as the coefficient of variation for duplicate measurements, was 1.6%.

### Measurement of polyamines

Cells were seeded in tissue culture dishes (6 cm diameter) in triplicate and, 24 hours later, cells were exposed to DENSPM (0.1 μM) or DFMO (50 μM). Cells were collected for polyamine analysis at the time of seeding and then 1 – 3 days after seeding. Briefly, cells were harvested, washed three times with ice-cold PBS and counted. Cell pellets were stored at -20º until analysis. Chromatographic separation and quantitative determination of the polyamines in cell extracts in 0.2 M perchloric acid were carried out using high-performance liquid chromatography (Hewlett-Packard 1100), with *O*-phtaldialdehyde as the reagent [48]. The experiment was performed twice.

### Statistics

All values report the mean ± standard error of the mean (SEM) unless otherwise specified. Differences between groups were analysed using ANOVA and then unpaired t-test (RS/1, Brooks Automation, Inc., Chelmsford, MA U.S.A.).

## Abbreviations

DENSPM: *N*^1^,*N*^11^-diethylnorspermine; DFMO: α-difluoromethylornithine; 5-FU: 5-fluorouracil; HNSCC: Squamous carcinoma cell line of the head and neck; ODC: Ornithine decarboxylase; SSAT: Spermine/spermidine *N*^1^-acetyltransferase.

## Ethical permission

Ethics Committee of Lund University (approval No. LU 376-01).

## Competing interests

The authors declare that they have no competing interests.

## Authors’contributions

JK participated in the design of the study, performed most of the experiments, and drafted the manuscript. JW conceived of the study, and participated in its design and coordination and helped to draft the manuscript. SMO helped in polyamine analysis and final drafting of the manuscript. All authors read and approved the final manuscript.
